# Viruses Associated with Acute Conjunctivitis in Vanuatu

**DOI:** 10.4269/ajtmh.22-0600

**Published:** 2023-01-16

**Authors:** Kasso Johnson, Fasihah Taleo, Kalbule Willie, Edwin Amel, Madopule Nanu, Marie Alguet, Jose Wass, Prudence Rymill, Anthony Solomon, Kevin Ruder, Cindi Chen, Lina Zhong, Armin Hinterwirth, David Liu, Thomas Abraham, Gerami Seitzman, Thomas Lietman, Thuy Doan, for the SCORPIO Study Group

**Affiliations:** ^1^ Ministry of Health Eye Program, Vanuatu;; ^2^ Vanuatu Country Office, World Health Organization, Port Vila, Vanuatu;; ^3^ Department of Control of Neglected Tropical Diseases, World Health Organization, Geneva, Switzerland;; ^4^ Francis I. Proctor Foundation, University of California San Francisco, San Francisco, California;; ^5^ Department of Ophthalmology, University of California San Francisco, San Francisco, California

## Abstract

The first manifestation of a viral infection may be conjunctivitis. There are limited data on the etiology of viral conjunctivitis in Vanuatu, a country in the South Pacific Ocean. Patients presenting to one of two Vanuatu health centers with presumed infectious conjunctivitis were eligible if symptom onset was within 14 days of screening. Conjunctival and anterior nasal swabs were obtained and subjected to unbiased RNA deep sequencing (RNA-seq) to identify DNA and RNA viruses. For samples collected from May to November 2021, RNA-seq identified a viral etiology in 12/48 patients. Human adenovirus species were the most common viruses (58%) detected, followed by human herpes viruses (cytomegalovirus, varicella zoster virus, and human herpes 7 virus). Rhinovirus C, Epstein-Barr virus, and bocavirus were also detected. In summary, the etiology for viral conjunctivitis in Vanuatu appears broad. Unbiased testing may be useful for disease surveillance.

Infectious conjunctivitis can be acute, new, and sudden in onset (less than 3 months) or chronic (3 months or more) and may result in significant ocular morbidity. Chronic infectious conjunctivitis secondary to the ocular strains of *Chlamydia trachomatis* (“trachoma”) disproportionally affects low-resource countries.^1^^,^^2^ Although Vanuatu has recently been declared the first Pacific Island nation to eliminate trachoma, very little is known about causes of acute conjunctivitis in this country, and there is a paucity of previously published data on the molecular or microbiological diagnosis of acute conjunctivitis in the Pacific Islands.^3^ Acute infectious conjunctivitis is generally heterogeneous and dependent on region, and affects all populations.^4^ Viral etiology, such as human adenovirus (HAdV), can be highly transmissible and has important public health implications, including lost wages due to time off work and the overprescription of topical antibiotics.^5^^,^^6^ SCORPIO (Seasonal Conjunctivitis Outbreak Reporting for Prevention and Improved Outcomes) is a collaborative study including over 20 international sites that leverages unbiased RNA deep sequencing (RNA-seq) to interrogate the global etiology of acute infectious conjunctivitis.^7^^,^^8^

This article focuses on the viral etiology of acute conjunctivitis in Vanuatu. SCORPIO was approved by the University of California San Francisco (UCSF) institutional review boards and the Vanuatu Ministry of Health. This study adhered to the tenets of the Declaration of Helsinki. The local study team consisted of Vanuatu’s practicing ophthalmologists, nurses, and healthcare workers who are trained in the identification of conjunctivitis. The entire study team participated in acute conjunctivitis study protocol training. Patients of any age with presumed infectious conjunctivitis of up to 14 days’ duration were prospectively enrolled, on the day of presentation, at two sites, Santo and Port Vila, in Vanuatu. Informed consent was obtained from all patients or guardians for children under 18 years of age. Sterile polyester applicators (Puritan, Guilford, ME) were used to swab the lower fornix of each eye and each anterior nasal passage. Swabs were placed in DNA/RNA-Shield media (Zymo Research, Irvine, CA) to inactive pathogens and preserve nucleic acids, and then stored in a −20°C freezer prior to shipping to UCSF for processing. Sample processing, library preparation, and sequencing have been previously described.^7^^,^^9^ The prespecified criteria for presumed pathogen identification were, in brief: 1) virus known to be a human pathogen and representing the most abundant matched reads after water background subtraction; 2) two or more unique reads covering separate regions in DNA virus genomes; or 3) one or more unique reads matching RNA virus genomes. All confidence intervals (CIs) were calculated using the adjusted Wald method.

From May to November 2021, we enrolled 48 patients. Of those 48 patients, samples from 12 (25%) tested positive for viral RNA fragments on unbiased RNA-seq. Patient demographics and clinical symptoms and signs from the 12 patients with viral conjunctivitis pathogens are shown in Table 1. Patients’ ages ranged from 1 month to 68 years; 42% (95% CI: 19–68%) were female. The mean time of symptom onset to presentation was 5 days (range: 1–14 days). Bilateral eye involvement occurred in 33% (95% CI: 14–61%). The most common systemic symptom was coughing (42%; 95% CI: 19–68%), followed by rhinorrhea (33%; 95% CI: 14–61%) and sore throat (9%; 95% CI: 0–40%). Forty-two percent (95% CI: 19–68%) of patients reported affected contacts or family members. On examination of the eyes, 91% (95% CI: 60–100%) presented with purulent discharge, 50% (95% CI: 25–75%) with tearing, 36% (95% CI: 15–65%) with subepithelial infiltrates, and 20% (95% CI: 5–52%) with membranes or pseudomembranes. No patients reported vomiting, although one patient reported diarrhea. No patients had preauricular lymphadenopathy on examination. Forty-five percent (95% CI: 21–72%) of patients presented on topical antibiotics.

**Table 1:** Patient demographics, clinical symptoms and signs, and associated viruses

Demographics				Clinical symptoms and signs												Medications and sequencing		
Patient no.	Age	Sex	Contact affected	Symptom duration (d)	Eye (s) affected	Sore throat	Runny nose	Coughing	Diarrhea	Itching	Preauricular lymphadenopathy	Tearing	Purulent discharge	Subepithelial infiltrates	Membrane or pseudomembrane	Topical medications	Results	Sites*
1	8 mo	M	No	1	Right	No	No	No	No	No	No	No	Yes	No	Unknown	No	CMV	N, OS
2	2 yr	M	No	7	Left	No	Yes	Yes	No	No	No	Yes	Yes	No	No	No	HAdV-B	N, OD, OS
3	7 yr	F	Yes	6	Both	No	No	No	No	No	No	No	Yes	No	No	Tetracycline	HAdV-B	OS
4	5 yr	M	Yes	5	Left	No	Yes	Yes	No	No	Unknown	Yes	Yes	Yes	Yes	No	HAdV-C	N
5	68 yr	F	No	7	Left	No	Yes	Yes	No	No	No	No	Yes	No	No	Tetracycline	VZV	N
6	47 yr	F	No	2	Right	No	No	No	No	No	No	Yes	Yes	Yes	Yes	Tetracycline	HAdV-B	N, OD, OS
7	7 yr	M	Yes	3	Right	Yes	Yes	Yes	No	Yes	No	Yes	Yes	No	No	Tetracycline	HAdV-B	OS
8	11 yr	F	Yes	14	Both	No	No	No	No	No	No	No	Yes	No	No	Ciprofloxacin	EBV	OS
9	3 yr	F	No	7	Both	No	No	No	No	No	No	Yes	Yes	Yes	No	No	Bocavirus	OD
10	3 yr	M	Yes	1	Left	Unknown	Yes	Yes	Yes	No	No	No	Yes	No	No	No	Rhinovirus C	N
11	1 mo	M	No	3	Both	No	No	No	No	No	No	Yes	No	Unknown	Both	No	HAdV-D	N, OD, OS
12	22 yr	M	No	3	Left	No	No	No	No	No	No	No	Unknown	Yes	Unknown	No	HAdV-B, HHV-7	OD

CMV = cytomegalovirus; EBV = Epstein-Barr virus; F = female; HAdV = human adenovirus; HHV-7 = human herpes virus 7; M = male; N = nasal; OD = right eye; OS = left eye; VZV = varicella zoster virus.

* Sample collection site in which the identified virus(es) met the pathogen call criteria.

Of the viruses identified as associated with conjunctivitis, RNA-seq demonstrated that DNA viruses were the most common pathogens (Figure 1). Of the DNA viruses, seven patients had detectable HAdV RNA in one of their samples taken from either the conjunctiva or nose. Other DNA viruses included cytomegalovirus (CMV), varicella zoster virus (VZV), Epstein-Barr virus (EBV), and bocavirus (Table 1, Figure 1). One patient had codetection of HAdV-B and human herpes virus 7 (HHV-7). Rhinovirus C, a nonenveloped, positive-strand RNA virus, was detected in a 3-year-old boy who had presented with coughing and diarrhea (Figure 2).

**Figure 1.**
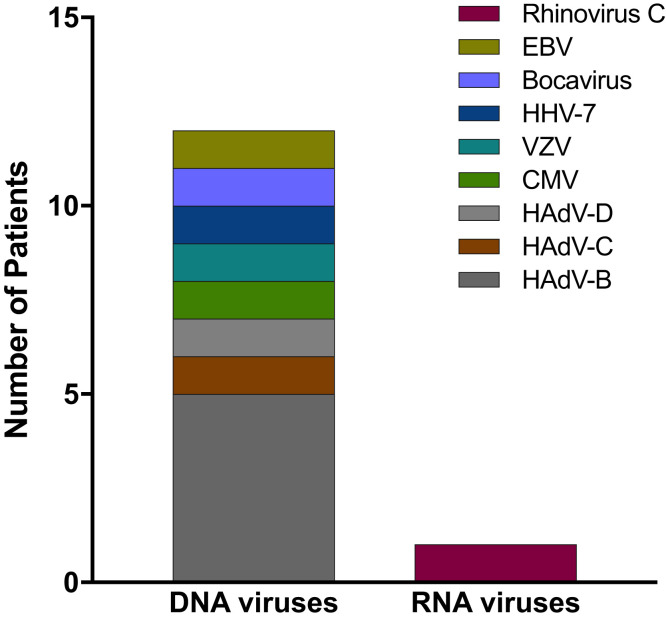
Stacked bar graph of DNA and RNA viruses detected with RNA-seq. CMV = cytomegalovirus; EBV = Epstein-Barr virus; HAdV = human adenovirus; HHV-7 = human herpes virus 7; VZV = varicella zoster virus.

**Figure 2.**
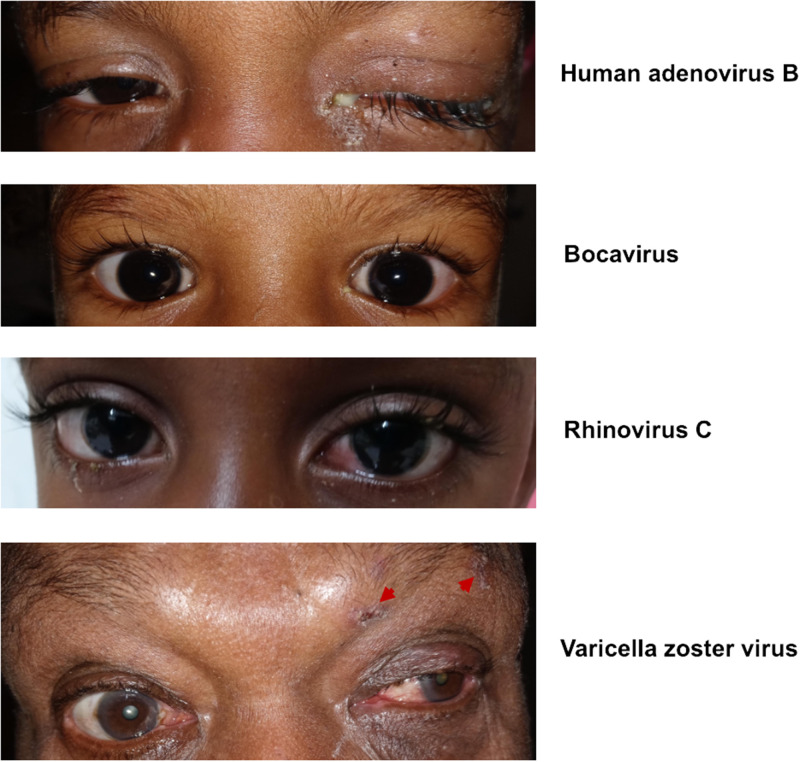
Representative external photos of affected patients. Top panel: 2-year-old boy with left eye involvement, purulent discharge, and eyelid crustiness associated with human adenovirus B. Second panel: 3-year-old girl with bilateral eye involvement associated with bocavirus. The left eye was more injected than the right eye with associated tearing and discharge. Third panel: 3-year-old boy with associated rhinovirus C infection. Bottom panel: 68-year-old man with left eye involvement associated with varicella zoster virus. Scabbing of vesicles (red arrows) can be seen in the V1 distribution.

In this cohort of patients from Vanuatu, we found that viral-associated conjunctivitis was identified in 25% (12/48) of patients who presented acutely to two health centers. Of those patients, HAdV species accounted for 58%. Although the majority of adenovirus cases were associated with species B, species D and C were also detected. The HAdV types included 5, 7, and 8, indicating that multiple strains were circulating in this population. Human herpes viruses (VZV, CMV, and HHV-7) comprised the second-largest group of pathogens detected.

The patient enrollment period corresponded to various waves of severe acute respiratory syndrome coronavirus 2 (SARS-CoV-2) transmission worldwide. However, Vanuatu did not experience any large outbreaks until January 2022. During the time of sample collection, Vanuatu had fewer than seven cases of SARS-CoV-2 infection documented.^10^ It was not surprising that no SARS-CoV-2 RNA was detected in any of the samples we collected.

Bocavirus was detected in a conjunctival sample of a 3-year-old girl with bilateral eye involvement. She was noted to have rhinorrhea, suggesting upper respiratory involvement. Bocaviruses belong to the family Parvoviridae, and are small, icosahedral, nonenveloped, single-stranded DNA viruses. They are frequently detected in the upper airways of children with respiratory symptoms and had been documented in children with conjunctivitis.^11^^,^^12^ Longitudinal serology analysis suggested that the majority of children had acquired bocavirus infection by 6 years of age and that primary infections are strongly associated with respiratory symptoms.^11^ It remains a debate whether bocaviruses are truly pathogenic, because they are frequently coidentified with other bacteria and viruses.^11^^,^^12^ Antibody testing for IgM was not assessed in this patient. However, RNA-seq detected bocavirus RNA and, given that this is a DNA virus, this indicated that actively replicating viral particles were present on the conjunctiva of this patient.

The only RNA virus detected in our cohort was rhinovirus C. Rhinoviruses are positive-strand RNA viruses in the *Enterovirus* genus. Rhinoviruses can be detected in the conjunctiva and upper and lower airways of patients with respiratory tract infections and in fecal samples of children with gastroenteritis.^13^^,^^14^ Our patient was a 3-year-old boy who presented with coughing, diarrhea, and conjunctivitis of the left eye. He had been exposed to a sick contact. He had an ocular purulent discharge, but was not noted to have corneal involvement.

The unbiased nature of RNA-seq allowed for the identification of multiple viruses in the same host. In a 22-year-old man, both HAdV-B and HHV-7 RNA fragments were detected in the conjunctival sample. Subepithelial infiltrates were noted in the affected eye, but visual acuity remained 20/20. Although HHV-7 can be associated with conjunctivitis, it is highly prevalent in persons older than 6 years of age and can be found to shed intermittently in saliva.^15^^,^^16^ Thus, we suggest that the patient’s ocular symptoms were likely secondary to HAdV-B infection. It was unclear whether detection of HHV-7 in this setting represented coinfection, reactivation, or colonization.^17^^,^^18^

Viral conjunctivitis has generally been attributed to human adenoviruses.^19^^,^^20^ Here, viral conjunctivitis represented only a quarter of all patients tested. However, HAdV infection accounted for the majority of viral pathogens identified, with HAdV-B7 predominant. HAdV-B7 is known to cause severe respiratory disease, epidemic keratoconjunctivitis, and acute hemorrhagic conjunctivitis.^21^^,^^22^ None of the patients reported severe respiratory disease, although some presented with mild upper respiratory symptoms such as coughing. Two patients with HAdV-B7 presented with corneal involvement. No hemorrhagic conjunctivitis was documented.

The main limitations of the study include the small sample size collected at the two health centers in Vanuatu and the lack of traditional microbiological testing. Worldwide, however, testing of any type for the etiology of conjunctivitis is rarely performed in the ambulatory setting. Additional limitations include the possibility that the patient cleared the pathogen prior to swabbing and that topical medications used at the time of swabbing limited pathogen detection. Finally, although there were only a few confirmed cases of SARS-CoV-2 in Vanuatu during the enrollment period, patients were anecdotally less likely to seek care than prior to the pandemic, and health centers were not operating at full capacity. Thus, the results reported in the study are likely to be an underestimation of the circulating viruses associated with infectious conjunctivitis in the population.

In summary, this study suggests that DNA viruses are commonly associated with acute conjunctivitis in Vanuatu. Multiple human adenovirus types can circulate at the same time and affect children and adults alike. It is unclear whether this pattern has evolved over time, because there exists limited literature on the viral etiology of conjunctivitis in Vanuatu. As SCORPIO continues to characterize pathogens causing acute conjunctivitis in Vanuatu and around the world, regional priorities for treatment pathways, surveillance, and diagnostic and therapeutic research may become clearer.
